# Effects of Alkaline-Reduced Drinking Water on Irritable Bowel Syndrome with Diarrhea: A Randomized Double-Blind, Placebo-Controlled Pilot Study

**DOI:** 10.1155/2018/9147914

**Published:** 2018-04-15

**Authors:** Dong Woo Shin, Hyuk Yoon, Hyun Soo Kim, Yoon Jin Choi, Cheol Min Shin, Young Soo Park, Nayoung Kim, Dong Ho Lee

**Affiliations:** ^1^Department of Internal Medicine and Seoul National University Bundang Hospital, Seongnam, Gyeonggi-do, Republic of Korea; ^2^Department of Internal Medicine and Liver Research Institute, Seoul National University College of Medicine, Seoul, Republic of Korea

## Abstract

**Objectives:**

The purpose of this study was to investigate whether the ingestion of alkaline-reduced water (ARW) is helpful in improving the symptoms of diarrhea-predominant irritable bowel syndrome (IBS).

**Methods:**

Twenty-seven patients (male, 25.9%; mean, 41.7 years old) with diarrhea-predominant IBS were randomly allocated to two groups. For eight weeks, the ARW group (*n* = 13) ingested at least 2 liters/day of ARW, while the control group (*n* = 14) ingested placebo water. IBS symptom scores (quality-of-life, abdominal pain/discomfort), stool form, and frequency were assessed before and after treatment via questionnaires.

**Results:**

Eight patients (61.5%) in the ARW group and six patients (42.9%) in the control group indicated that their symptoms had improved in more than four out of the eight weeks of treatment (*p* = 0.449). The IBS quality-of-life score significantly improved from 57.2 to 30.8 in the ARW group; this improvement was significantly greater than the slight improvement from 48.7 to 42.2 observed in the control group (*p* = 0.029). The abdominal pain score improved from 1.8 to 0.9 in the ARW group and from 1.8 to 1.1 in the control group, with no significant group difference (*p* = 0.232).

**Conclusions:**

Drinking ARW for eight weeks improves the quality of life in patients with diarrhea-predominant IBS.

## 1. Introduction

Irritable bowel syndrome (IBS) is a functional intestinal disorder accompanied by abdominal pain and bowel habit changes, without evidence of an underlying injury. It is a very common disease, occurring in about 11% of people worldwide [1]. According to the Korean National Health Insurance System database, 5.1% of men and 6.9% of women were diagnosed with IBS [2]. IBS is one of the most common illnesses in primary care, with a repeated cycle of deterioration and relief over the years. Improving symptoms through appropriate treatment is important; IBS lowers the quality of life and increases medical costs [2–4]. Patients with IBS also suffer from anxiety, major depressive disorder, and chronic fatigue syndrome [5, 6]. However, the cause and mechanisms underlying the various symptoms are not entirely understood. Many hypotheses have been proposed, including small bowel bacterial overgrowth syndrome, genetic factors, food hypersensitivities, gastrointestinal motility disorders, gut-brain axis alterations, hypersensitivity of the intestine, and psychosocial factors [7–10]. Recent studies indicate that the intestinal microbiota is one of the important factors affecting the onset of IBS [11, 12].

Various drugs have been used to improve symptoms, including antacids, antispasmodics, and drugs that stimulate gastrointestinal motility (prokinetic agents). However, with a lack of convincing evidence for a pathophysiological basis, conventional therapies have not achieved complete symptom improvement. Therefore, several alternative therapeutic methods, including dietary changes, probiotics, and other medications, have been proposed [13–16]. Furthermore, mineral water with various electrolyte compositions has been utilized in the treatment of functional gastrointestinal diseases; mineral water supplements have been reported to improve functional dyspepsia associated with IBS by controlling gastric acid output and intestinal transit time [17]. In addition, carbonated water not only attenuates the hunger but also improves dyspeptic symptoms and heartburn [18, 19]. Drinking sulfur-rich mineral water for more than three weeks was found to be effective in treating constipation by increasing frequency of bowel movements [20]. Bicarbonate-containing alkaline-reduced water (ARW) has also been hypothesized to affect various digestive functions. Although animal studies have provided evidence that ARW is effective in treating functional bowel disease, human studies are lacking [21–23]. Therefore, the purpose of this randomized double-blind pilot study was to evaluate the effect of ARW ingestion on diarrhea-predominant IBS.

## 2. Methods

### 2.1. Ethical Approval

This study was conducted in accordance with the ethical principles for medical research involving human subjects in the Declaration of Helsinki. This study was approved by the Seoul National University Bundang Hospital Medical Ethics Committee (IRB number: E-1405/250-002) and aspires to protect the lives, health, privacy, and dignity of the research participants. Thus, the purpose and characteristics of the clinical trial were fully explained to the participants. Only patients who voluntarily signed an informed consent were included, and patients were allowed to stop participating at any time during the trial. All results obtained in this clinical study are confidential.

### 2.2. Study Population

Men and women aged 18–75 years who met Rome III criteria [24] for diarrhea-predominant IBS, had no underlying disease of the colon on a sigmoidoscopy or colonoscopy performed within 5 years prior to screening, and could understand and respond to the symptom questionnaires were included. Rome III criteria for IBS involve recurrent abdominal pain or discomfort at least 3 days/month in the last 3 months with two or more of the following: improvement with defecation, onset associated with a change in stool frequency, or onset associated with a change in stool form [24]. Diarrhea-predominant IBS involves loose or watery stools in more than 25% of bowel movements and hard or lumpy stool in less than 25% of bowel movements.

The following were excluded: patients with a psychiatric history; patients with untreated malignant tumors; patients with severe liver or kidney disease (AST, ALT levels 3-fold greater than the normal upper limit, and serum creatinine levels 1.5-fold greater than the normal upper limit); patients with severe heart failure; patients with acute gastrointestinal tract infection within the last 3 months. In addition, patients who were taking medications during the study period that could affect the results were also excluded. This included drugs that might influence IBS symptoms, such as antispasmodics, laxatives, prokinetics, anticholinergics, antianxiety drugs, antidepressants, analgesics, thyroid hormone, antibiotics, and steroids.

It is difficult to predict the therapeutic response rate between the test group and the control group since similar studies related to ARW have not existed before. This is a small-scale preliminary pilot study to investigate feasibility, adverse events, and improvement before a full-scale research project. This study was planned with 30 participants per group, which is the minimum number of participants recommended in a pilot study [25, 26]. Given an estimated dropout rate of 15%, at least 35 people per group were planned to be enrolled.

### 2.3. Randomization and Allocation

Patients who were diagnosed with diarrhea-predominant IBS by Rome III criteria were equally allocated to experimental and control groups. Randomization was performed using a 1 : 1 computerized block randomization with a predetermined random code. Because both the investigator and the patients were blinded, a research coordinator performed the random assignment. The research coordinator did not provide information on randomization to the patients and researchers until the end of the study. Neither the participants nor the researchers could distinguish group assignments.

### 2.4. Study Design

**Figure 1:**
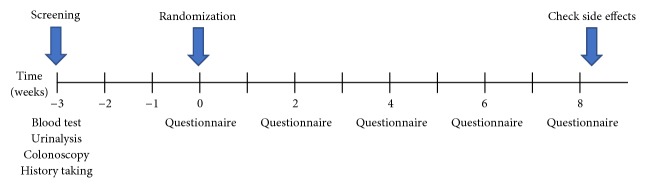
Schedule of patients participating in the study.

A flowchart of the study design is provided in Figure 1. Patients completed screening tests (blood, urinalysis, colonoscopy, and a past medical history questionnaire) 1–3 weeks before participating in the study. Laboratory evaluation included assessments of liver function (albumin, total bilirubin, aspartate aminotransferase, alanine aminotransferase, and alkaline phosphatase levels), kidney function (creatinine and blood urea nitrogen levels), electrolytes (sodium, potassium, chloride, calcium, and inorganic phosphorus levels), and the complete blood count (CBC).

Baseline questionnaires on the IBS quality of life, abdominal pain/discomfort, stool form, and stool frequency were completed at the start of the study. The IBS quality-of-life questionnaire is an indicator of abdominal discomfort and consists of 34 items (each recorded as 1–5 points: 1: not at all, 2: slightly, 3: moderately, 4: quite a bit, and 5: extremely) [27]. Symptom scores for abdominal pain/discomfort were rated on a scale of 0–4 (0: asymptomatic, 1: mild, 2: moderate, 3: severe, and 4: very severe) and were based on the worst level of the day. Abdominal discomfort was defined as an uncomfortable sensation not described as pain. Stool form was assessed using the Bristol stool scale, which is a diagnostic tool designed to classify the form of human feces into seven categories. In general, types 1 and 2 (hard or lumpy stool) indicate constipation, and types 5–7 (soft or watery stool) indicate diarrhea [25]. In addition, the number of bowel movements was recorded daily.

The experimental group ingested ARW from an installed test device, while the control group ingested placebo water from a sham device. Both groups were instructed to ingest more than 2 liters per day for eight weeks. Participants visited the hospital every two weeks and completed self-administered questionnaires on compliance, adverse effects, the amount ingested, symptom scores (abdominal pain/discomfort), stool form, and the number of daily bowel movements. Questionnaires on the IBS quality of life were completed only at the end of the eighth week. If adverse events occurred during the trial period, participants were instructed to stop the medication immediately and visit an outpatient clinic.

The primary outcome was the proportion of participants with adequate symptomatic improvement in more than four weeks of the 8-week treatment period. The secondary outcomes were changes in IBS quality of life, symptom scores (abdominal pain/discomfort), and stool form/frequency.

### 2.5. Research Equipment

ARW with a pH of 8.5–10.0 was produced using an alkali water ionizer (Kim Young Kwi alkali water ionizer, KYK33000). Placebo water was prepared using a sham device (model name: sham KYK33000), which was not able to generate ARW, but had the same appearance as that of the test apparatus. The devices were installed at the patient's home and patients were allowed to drink water as needed.

### 2.6. Statistical Analyses

Statistical analyses were performed using SPSS for Windows (ver. 22.0, IBM Corporation, Chicago, IL, USA) and STATA software (ver. 14.0, STATA Corporation, College Station, TX, USA). Group differences were evaluated using Student's *t*-test for continuous variables and the Chi-square or Fisher's exact test for categorical variables. Group differences in treatment-related changes in variables related to IBS (abdominal pain/frequency, stool form, and frequency of bowel movements) were evaluated using a linear mixed model with an interaction term between group and time (before and after treatment). Changes in the IBS quality-of-life score were evaluated using the paired *t*-test. Two-sided *p* values less than 0.05 were considered statistically significant.

## 3. Results

### 3.1. Baseline Characteristics

**Figure 2:**
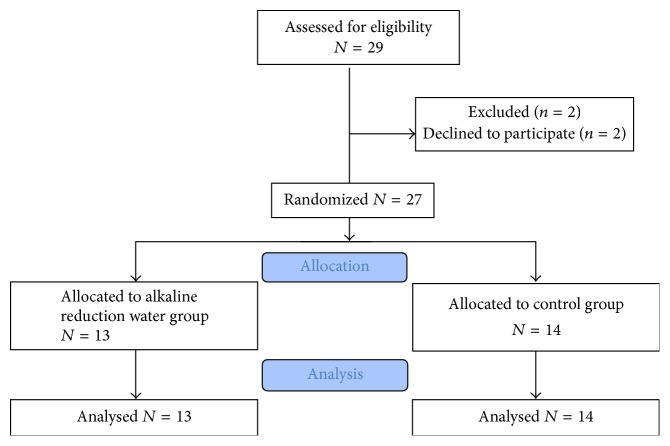
CONSORT flow diagram of patient recruitment.

 Only 29 were enrolled in the study and 2 dropped out during the study; because the patients were burdened with drinking more than 2 liters of water a day for a long time, we failed to enroll the intended 70 patients. Finally, 13 patients in the ARW group and 14 patients in the control group completed the study (Figure 2). There were no significant group differences in baseline characteristics (Table 1). Ten out of thirteen (76.9%) patients in the ARW group and ten out of fourteen (71.4%) patients in the control group were women. The mean age in the ARW group was slightly higher compared to that of the control group, but without statistical significance (43.3 versus 40.1, *p* = 0.584). At the beginning of the study, IBS symptom scores (quality-of-life, abdominal pain/discomfort), Bristol stool form, and stool frequency were not significantly different between the two groups. In addition, the consumption of water was similar in the two groups (ARW group: 2,124 ± 900 ml/day; control group: 2,052 ± 648 ml/day, *p* = 0.834).

### 3.2. Primary Outcome Measure

Table 2 shows the number of responders (a favorable symptom improvement in more than four weeks of the eight-week treatment period) and nonresponders in each group. Although the proportion of patients responding to the treatment was higher in the ARW group (8/13, 61.5%) than in the control group (6/14, 42.9%), the difference was not statistically significant (Fisher's exact test, *p* = 0.449).

### 3.3. Secondary Outcome Measures

**Figure 3:**
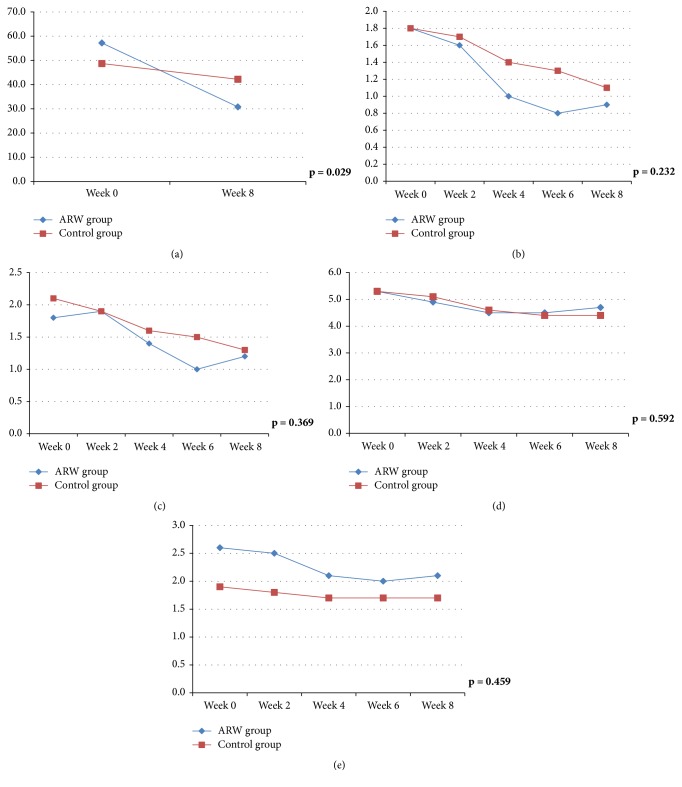
Graph of change before and after treatment of IBS. (a) Quality-of-life score. (b) Abdominal pain score. (c) Abdominal discomfort score. (d) Bristol stool form scale. (e) Stool frequency per day.

After eight weeks of treatment, the IBS quality-of-life score had improved from 57.2 to 30.8 points in the ARW group and from 48.7 to 42.2 in the control group (Table 3), with a significant group difference (Figure 3(a), *p* = 0.029). The abdominal pain score improved from 1.8 to 0.9 in the ARW group and from 1.8 to 1.1 in the control group, without a statistically significant group difference (Figure 3(b), *p* = 0.232). Abdominal discomfort, stool form, and stool frequency were somewhat improved in the ARW group; however, there were no significant group differences (Figures 3(c)–3(e)).

### 3.4. Adverse Effects

One of the patients in the control group visited the emergency room due to vomiting and abdominal pain during the second week of the study, but improved with conservative treatment. There were no specific adverse effects associated with ARW ingestion during the eight weeks of the trial.

## 4. Discussion

IBS is one of the most common gastrointestinal disorders in the general population [1]. In addition, because the effects of medications are often temporary, patients may increase the dose of the medication or take several medications, resulting in the occurrence of side effects. Thus, interest in alternative therapies that do not have side effects (even after long-term use) is growing [13–17]. Numerous animal studies have investigated the ability of controlling the electrolyte balance or acidity of the drinking water to treat functional gastrointestinal disorders. For example, animal studies have shown ARW to be effective in treating gastritis because it permanently denatures pepsin [21]. In addition, an animal study demonstrated that ingestion of more than 1.5 liters of bicarbonate-alkaline mineral water for 30 days improves dyspeptic symptoms [22]. It has also been suggested that a regular course of crenotherapy with bicarbonate-alkaline mineral water can be used to treat functional dyspepsia, improving gastrointestinal motility and secretory function by modulating the secretion of peptide hormones and regulating the movement of digestive organs [23]. These studies support the hypothesis that ARW can effectively treat IBS; however, prior to the present study, there were no supporting human clinical trials. Given this preclinical basis, we aimed to investigate whether ARW ingestion for eight weeks improved the symptoms of IBS.

This randomized controlled, double-blind, placebo-controlled study was designed to determine whether the ingestion of ARW could improve the quality of life, abdominal pain/discomfort, stool form, and stool frequency in diarrhea-predominant IBS. In terms of the primary endpoint, the proportion of responders (IBS patients who had improved symptoms in more than four weeks of the 8-week treatment period) was higher in the ARW group than in the control group, but the group difference was not statistically significant. This is likely due to the small number of patients who completed the trial; however, it is hard to predict an effect size, as no similar studies exist. We believe that a positive result could be obtained in a larger-scale study. In contrast to the primary outcome, a significant group difference was observed in the secondary outcomes. The IBS-related quality-of-life and abdominal pain scores were decreased to a greater extent with ARW ingestion compared to those with the ingestion of placebo water. This is a meaningful result because it demonstrates that it is possible to reduce IBS symptoms simply by ingesting water with a different pH, without taking any other medication. In addition, ARW has few adverse effects; thus, it shows potential in becoming an important complementary therapy for functional bowel disease. However, there were no significant group differences in the stool form and frequency improvements. At the beginning of the study, the frequency of bowel movements in both groups was 2-3 times a day, which is less than that for the definition of diarrhea (more than three times a day). Thus, the patients in both groups mainly had mild diarrhea, which may explain the lack of a significant change in symptom scores with treatment.

The mechanism by which ARW improves IBS symptoms remains unclear. ARW refers to water with a pH of at least 8.4; in contrast, most tap or bottled water has a pH between 6.7 and 7.4 [21]. ARW is thought to increase the pH level of the stomach given its large amount of bicarbonate ions. Interestingly, just infusing a small amount (0.1 mol/L) of acid into the stomach can aggravate indigestion in most people [28]. In addition, acidification of the duodenum exacerbates dyspeptic symptoms by inducing proximal gastric relaxation and inhibiting gastric accommodation to a meal [29]. In one animal study, duodenal acidification-induced gastric hypersensitivity could be the cause of dyspepsia in patients with IBS and serotonin 5-HT3 receptors play a key role [30]. Furthermore, in patients with pancreatic insufficiency, such as cystic fibrosis, the small intestine is exposed to an acidic environment, resulting in impaired absorption. Rapid neutralization of gastric acid in the proximal portion of the duodenum and tight regulation of the gastrointestinal pH play important roles in maintaining nutrient absorption and function in the intestines [31]. In addition, mineral water with a unique electrolyte composition may help improve the symptoms of indigestion [18–20, 32]. Carbonated water could regulate gastrointestinal motility diseases by stimulating bile flow and pancreatic exocrine secretion. Furthermore, drinking carbonated water for more than 15 days has been shown to improve gallbladder muscle contractions [32]. The ingestion of water containing a lot of mineral salts has been shown to improve gastric emptying in patients with indigestion [33]. It is presumed that the various ions contained in mineral water directly or indirectly (via neuroendocrine secretion of vasointestinal peptides) stimulate the smooth muscle involved in gastrointestinal motility. These actions appear to improve the symptoms of IBS by improving intestinal transit time and excretory capacity. These actions are thought to not only reduce the bowel transit time, but also promote gastrointestinal hormone secretion, thereby improving abdominal bloating. We expect that large-scale studies on ARW with various electrolytic compositions will proceed in the future.

Gut microbiota appear to be one of the important factors contributing to the cause and pathophysiology of IBS [11, 12]. Postinfectious IBS should be suspected when the patient complains of dyspepsia or abdominal discomfort after acute gastroenteritis [34]. Postinfectious IBS is thought to be due to persistent low-grade inflammation and alteration of gut flora intestinal microorganisms. The composition of gut microbiota is also associated with the pathophysiology of IBS and the host immune response [35]. Abundance of* Cyanobacteria* is associated with bloating, satiety, and increased abdominal discomfort. The amount of* Proteobacteria* is associated with pain threshold [36]. Therefore, it was suggested that probiotics, antibiotics, and fecal microbiota transplantation might be effective in the treatment of IBS [37]. Many gastrointestinal disorders, including IBS, are caused by an imbalance of residential microflora of the intestinal tract. Human intestinal microbiota consist of 96–99% anaerobes and 1–4% aerobes. Microorganisms have their own intrinsic reduction potential* (Eh)* for each species, and aerobic and anaerobic bacteria grow at different oxidation-reduction potentials. Aerobic bacteria require a positive potential of +400 mV and facultative anaerobic bacteria require negative electric potential between −300 and −400 mV. Electrochemically generated reduced water has a negative potential of 0 to −300 mV, while the tap water has a potential of +300 to +450 mV [38]. By drinking reduced water, it is possible to improve symptoms of functional bowel disease by accelerating the growth of anaerobic bacteria (*Lactobacilli* and* Bifidobacteria*) and inhibiting the growth of aerobic pathogens.

The present study has some limitations. First, the statistical power was weak because of the small sample size. Second, patients with IBS tend to be somewhat less adherent due to the distrust of conventional therapies and hospitals. Third, although IBS is a highly prevalent disease, there were some difficulties in recruiting patients. Because the participants expressed difficulty in drinking more than 2 liters of water a day, we could not enroll as many patients as intended. In addition, subjects were already taking several medications before participating in the study, so it was not easy to stop them all and treat them with ARW only for 8 weeks. Patients' compliance should be taken into account when designing large-scale studies on this topic in the future. Fourth, the lifestyle and diet were not controlled except for the medications. These confounding factors may be somewhat offset in the randomization process. Despite these limitations, the main strength of the present study is its randomized, double-blind, placebo-controlled design. Moreover, ARW is a simple and inexpensive treatment that physicians can easily consider in the treatment of IBS. To our knowledge, this is the first study to show whether ARW can improve IBS in humans, irrespective of the mechanism.

In conclusion, the present study suggests that ingestion of ARW can improve the quality of life and reduce abdominal pain in patients with diarrhea-predominant IBS. We hope that this pilot study provides a cornerstone for future large-scale trials on the effectiveness of ARW in the treatment of IBS.

## Figures and Tables

**Table 1:** Characteristics of baseline demographics of patients.

	Alkaline-reduced water group (*n* = 13)	Control group (*n* = 14)	*p* value
Female, *n* (%)	10 (76.9%)	10 (71.4%)	0.745
Mean age ± SD (years)	43.3 ± 14.4	40.1 ± 15.7	0.584
Initial symptom scores			
Quality-of-life score	57.2 ± 28.0	48.7 ± 26.4	0.428
Abdominal pain	1.8 ± 0.9	1.8 ± 0.8	0.983
Abdominal discomfort	1.8 ± 0.8	2.1 ± 0.8	0.362
Stool form (BSFS)	5.3 ± 0.5	5.3 ± 1.4	0.939
Stool frequency/day	2.6 ± 1.2	1.9 ± 1.0	0.130
Amount of water (ml/day)	2,124 ± 900	2,052 ± 648	0.834

SD: standard deviation; BSFS: Bristol stool form scale.

**Table 2:** Proportion of responders who showed symptomatic improvement after treatment (primary outcome measure).

	Alkaline-reduced water group (*n* = 13)	Control group (*n* = 14)	*p* value
Responder, *n* (%)	8 (61.5%)	6 (42.9%)	0.449
Nonresponder, *n* (%)	5 (38.5%)	8 (57.1%)	

**Table 3:** Symptom scores of patients before and after treatment (secondary outcome measures).

		Alkaline-reduced water group (*n* = 13)	Control group (*n* = 14)	*p* value
Quality-of-life score	Week 0	57.2 ± 28.0	48.7 ± 26.4	0.428
	Week 8	30.8 ± 24.9	42.2 ± 36.3	0.353

Abdominal pain	Week 0	1.8 ± 0.9	1.8 ± 0.8	0.983
	Week 2	1.6 ± 1.0	1.7 ± 0.8	0.796
	Week 4	1.0 ± 0.9	1.4 ± 0.8	0.324
	Week 6	0.8 ± 0.8	1.3 ± 0.7	0.123
	Week 8	0.9 ± 0.8	1.1 ± 0.6	0.480

Abdominal discomfort	Week 0	1.8 ± 0.8	2.1 ± 0.8	0.362
	Week 2	1.9 ± 1.1	1.9 ± 0.7	0.964
	Week 4	1.4 ± 1.2	1.6 ± 0.8	0.688
	Week 6	1.0 ± 0.7	1.5 ± 0.8	0.113
	Week 8	1.2 ± 0.9	1.3 ± 0.7	0.777

Stool form (BSFS)	Week 0	5.3 ± 0.5	5.3 ± 1.4	0.939
	Week 2	4.9 ± 0.8	5.1 ± 0.8	0.546
	Week 4	4.5 ± 0.8	4.6 ± 1.0	0.791
	Week 6	4.5 ± 0.8	4.4 ± 1.3	0.747
	Week 8	4.7 ± 0.9	4.4 ± 1.0	0.313

Stool frequency/day	Week 0	2.6 ± 1.2	1.9 ± 1.0	0.130
	Week 2	2.5 ± 1.1	1.8 ± 0.7	0.073
	Week 4	2.1 ± 0.9	1.7 ± 0.7	0.198
	Week 6	2.0 ± 0.8	1.7 ± 0.9	0.349
	Week 8	2.1 ± 0.9	1.7 ± 0.7	0.213

Week 0: the time of randomization; BSFS: Bristol stool form scale.

## References

[1] Canavan C., West J., Card T. (2014). The epidemiology of irritable bowel syndrome. *Journal of Clinical Epidemiology*.

[2] Jung H.-K., Kim Y. H., Park J. Y. (2014). Estimating the burden of irritable bowel syndrome: Analysis of a nationwide korean database. *Journal of Neurogastroenterology and Motility*.

[3] Canavan C., West J., Card T. (2014). Review article: The economic impact of the irritable bowel syndrome. *Alimentary Pharmacology & Therapeutics*.

[4] Gudleski G. D., Satchidanand N., Dunlap L. J. (2017). Predictors of medical and mental health care use in patients with irritable bowel syndrome in the United States. *Behaviour Research and Therapy*.

[5] Fond G., Loundou A., Hamdani N. (2014). Anxiety and depression comorbidities in irritable bowel syndrome (IBS): a systematic review and meta-analysis. *European Archives of Psychiatry and Clinical Neurosciences*.

[6] Janssens K. A. M., Zijlema W. L., Joustra M. L., Rosmalen J. G. M. (2015). Mood and anxiety disorders in chronic fatigue syndrome, fibromyalgia, and irritable bowel syndrome: Results from the LifeLines cohort study. *Psychosomatic Medicine*.

[7] Ghoshal U. C., Srivastava D. (2014). Irritable bowel syndrome and small intestinal bacterial overgrowth: meaningful association or unnecessary hype. *World Journal of Gastroenterology*.

[8] El-Salhy M. (2015). Recent developments in the pathophysiology of irritable bowel syndrome. *World Journal of Gastroenterology*.

[9] Wouters M. M., Vicario M., Santos J. (2016). The role of mast cells in functional GI disorders. *Gut*.

[10] Tanaka Y., Kanazawa M., Palsson O. S. (2018). Increased Postprandial Colonic Motility and Autonomic Nervous System Activity in Patients With Irritable Bowel Syndrome: A Prospective Study. *Journal of Neurogastroenterology and Motility*.

[11] Moloney R. D. (2016). Stress and the microbiota brain axis in visceral pain: relevance to irritable bowel syndrome. *CNS neuroscience & therapeutics*.

[12] Ringel Y., Ringel-Kulka T. (2015). The intestinal microbiota and irritable bowel syndrome. *Journal of Clinical Gastroenterology*.

[13] Whelan K., Martin L. D., Staudacher H. M., Lomer M. C. (2018). The low FODMAP diet in the management of irritable bowel syndrome: an evidence-based review of FODMAP restriction, reintroduction and personalisation in clinical practice. *Journal of Human Nutrition and Dietetics*.

[14] Moayyedi P., Mearin F., Azpiroz F. (2017). Irritable bowel syndrome diagnosis and management: A simplified algorithm for clinical practice. *United European Gastroenterology Journal*.

[15] Harper A., Naghibi M., Garcha D. (2018). The Role of Bacteria, Probiotics and Diet in Irritable Bowel Syndrome. *Foods*.

[16] Harris L. A., Baffy N. (2017). Modulation of the gut microbiota: a focus on treatments for irritable bowel syndrome. *Postgraduate Medical Journal*.

[17] Gasbarrini G., Candelli M., Graziosetto R. G., Coccheri S., Di Iorio F., Nappi G. (2006). Evaluation of thermal water in patients with functional dyspepsia and irritable bowel syndrome accompanying constipation. *World Journal of Gastroenterology*.

[18] Suzuki M., Mura E., Taniguchi A., Moritani T., Nagai N. (2017). Oral carbonation attenuates feeling of hunger and gastric myoelectrical activity in young women. *Journal of Nutritional Science and Vitaminology*.

[19] Pohl U., Auinger A., Bothe G., Uebelhack R. (2016). Pilot Trial on the Efficacy and Safety of a Natural Mineral Water Rich in Hydrogen Carbonate on Functional Dyspepsia and Heartburn. *Open Journal of Gastroenterology*.

[20] Naumann J., Sadaghiani C., Alt F., Huber R. (2016). Effects of Sulfate-Rich Mineral Water on Functional Constipation: A Double-Blind, Randomized, Placebo-Controlled Study. *Forschende Komplementärmedizin*.

[21] Koufman J. A., Johnston N. (2012). Potential benefits of pH 8.8 alkaline drinking water as an adjunct in the treatment of reflux disease. *Annals of Otology, Rhinology & Laryngology*.

[22] Nassini R., Andrè E., Gazzieri D. (2010). A bicarbonate-alkaline mineral water protects from ethanol-induced hemorrhagic gastric lesions in mice. *Biological & Pharmaceutical Bulletin*.

[23] Bertoni M., Oliveri F., Manghetti M. (2002). Effects of a bicarbonate-alkaline mineral water on gastric functions and functional dyspepsia: A preclinical and clinical study. *Pharmacological Research*.

[24] Drossman D. A., Dumitrascu D. L. (2006). Rome III: new standard for functional gastrointestinal disorders. *Journal of Gastrointestinal and Liver Diseases*.

[25] Lancaster G. A., Dodd S., Williamson P. R. (2004). Design and analysis of pilot studies: recommendations for good practice. *Journal of Evaluation in Clinical Practice*.

[26] Browne R. H. (1995). On the use of a pilot sample for sample size determination. *Statistics in Medicine*.

[27] Drossman D. A., Patrick D. L., Whitehead W. E. (2000). Further validation of the IBS-QOL: a disease-specific quality-of-life questionnaire. *American Journal of Gastroenterology*.

[28] Miwa H., Nakajima K., Yamaguchi K. (2007). Generation of dyspeptic symptoms by direct acid infusion into the stomach of healthy Japanese subjects. *Alimentary Pharmacology & Therapeutics*.

[29] Ishii M., Kusunoki H., Manabe N. (2010). Duodenal hypersensitivity to acid in patients with functional dyspepsia-pathogenesis and evaluation. *Journal of Smooth Muscle Research*.

[30] Nakata-Fukuda M., Hirata T., Keto Y., Yamano M., Yokoyama T., Uchiyama Y. (2014). Inhibitory effect of the selective serotonin 5-HT3 receptor antagonist ramosetron on duodenal acidification-induced gastric hypersensitivity in rats. *European Journal of Pharmacology*.

[31] Gelfond D., Ma C., Semler J., Borowitz D. (2013). Intestinal pH and gastrointestinal transit profiles in cystic fibrosis patients measured by wireless motility capsule. *Digestive Diseases and Sciences*.

[32] Cuomo R., Grasso R., Sarnelli G. (2002). Effects of carbonated water on functional dyspepsia and constipation. *European Journal of Gastroenterology & Hepatology*.

[33] Anti M., Lippi M. E., Santarelli L., Gabrielli M., Gasbarrini A., Gasbarrini G. (2004). Effects of mineral-water supplementation on gastric emptying of solids in patients with functional dyspepsia assessed with the 13C-octanoic- acid breath test. *Hepato-Gastroenterology*.

[34] Grover M., Camilleri M., Smith K., Linden D. R., Farrugia G. (2014). Postinfectious irritable bowel syndrome: Mechanisms related to pathogens. *Neurogastroenterology & Motility*.

[35] Joo Y.-E. (2015). Alteration of fecal microbiota in patients with postinfectious irritable bowel syndrome. *Journal of Neurogastroenterology and Motility*.

[36] Jeffery I. B., O'Toole P. W., Öhman L. (2012). An irritable bowel syndrome subtype defined by species-specific alterations in faecal microbiota. *Gut*.

[37] Dupont H. L. (2014). Review article: Evidence for the role of gut microbiota in irritable bowel syndrome and its potential influence on therapeutic targets. *Alimentary Pharmacology & Therapeutics*.

[38] Vorobjeva N. V. (2005). Selective stimulation of the growth of anaerobic microflora in the human intestinal tract by electrolyzed reducing water. *Med Hypotheses*.

